# Assessing Gibberellins Oxidase Activity by Anion Exchange/Hydrophobic Polymer Monolithic Capillary Liquid Chromatography-Mass Spectrometry

**DOI:** 10.1371/journal.pone.0069629

**Published:** 2013-07-26

**Authors:** Ming-Luan Chen, Xin Su, Wei Xiong, Jiu-Feng Liu, Yan Wu, Yu-Qi Feng, Bi-Feng Yuan

**Affiliations:** 1 Key Laboratory of Analytical Chemistry for Biology and Medicine (Ministry of Education), Department of Chemistry, Wuhan University, Wuhan, China; 2 College of Life Sciences, Wuhan University, Wuhan, China; RIKEN PSC, Japan

## Abstract

Bioactive gibberellins (GAs) play a key regulatory role in plant growth and development. In the biosynthesis of GAs, GA3-oxidase catalyzes the final step to produce bioactive GAs. Thus, the evaluation of GA3-oxidase activity is critical for elucidating the regulation mechanism of plant growth controlled by GAs. However, assessing catalytic activity of endogenous GA3-oxidase remains challenging. In the current study, we developed a capillary liquid chromatography – mass spectrometry (*c*LC-MS) method for the sensitive assay of *in-vitro* recombinant or endogenous GA3-oxidase by analyzing the catalytic substrates and products of GA3-oxidase (GA_1_, GA_4_, GA_9_, GA_20_). An anion exchange/hydrophobic poly([2-(methacryloyloxy)ethyl]trimethylammonium-*co*-divinylbenzene-*co*-ethylene glycol dimethacrylate)(META-*co*-DVB-*co*-EDMA) monolithic column was successfully prepared for the separation of all target GAs. The limits of detection (LODs, Signal/Noise = 3) of GAs were in the range of 0.62–0.90 fmol. We determined the kinetic parameters (*K*_m_) of recombinant GA3-oxidase in *Escherichia coli* (*E. coli*) cell lysates, which is consistent with previous reports. Furthermore, by using isotope labeled substrates, we successfully evaluated the activity of endogenous GA3-oxidase that converts GA_9_ to GA_4_ in four types of plant samples, which is, to the best of our knowledge, the first report for the quantification of the activity of endogenous GA3-oxidase in plant. Taken together, the method developed here provides a good solution for the evaluation of endogenous GA3-oxidase activity in plant, which may promote the in-depth study of the growth regulation mechanism governed by GAs in plant physiology.

## Introduction

Gibberellins (GAs), one family of acidic phytohormones, play crucial roles in a number of plant growth and developmental processes, such as seed germination, stem elongation, leaf expansion, and flower development [1], [2]. So far, although the number of GAs identified from plants has exceed a hundred, only several GAs, including GA_1_, GA_3_, GA_4_ and GA_7_, are considered to function as bioactive plant hormones [3], [4]. Previous reports suggest that endogenous developmental cues and environmental signals can affect GAs-controlled plant growth by modulating GAs biosynthesis [5], [6]. Therefore, investigation of the regulation of bioactive GAs biosynthesis is crucial for revealing the mechanism of how these hormones control plant developmental processes.

**Figure 1:**
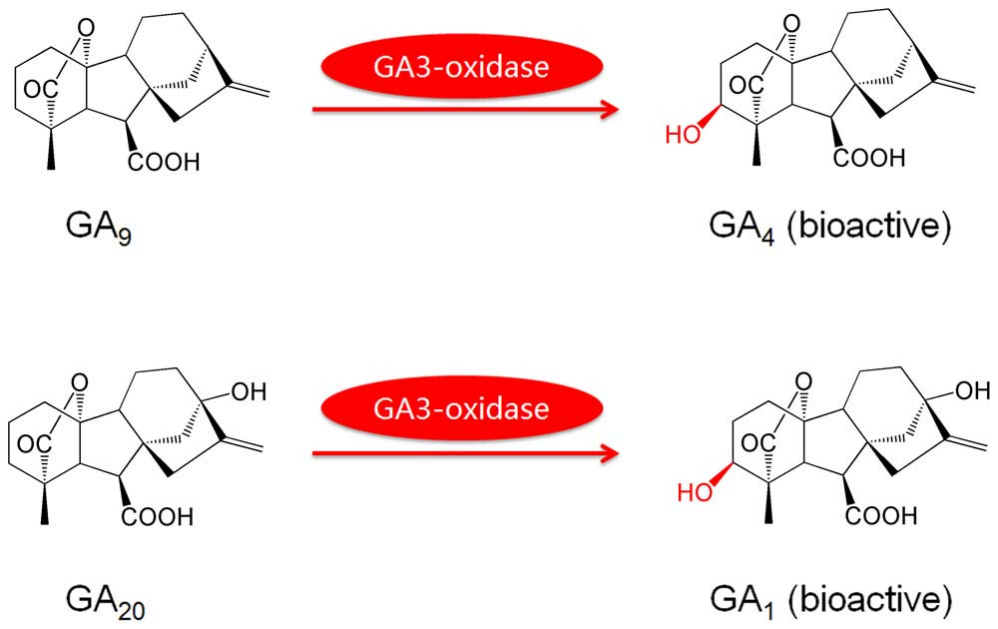
The reactions catalyzed by GA3-oxidase. In each metabolic reaction, the modification is highlighted in red.

GAs are biosynthesized through an intricate pathway that involves several kinds of enzymes. Recently, the discovery that the GA3-oxidase possesses high catalytic activity in *Arabidopsis thaliana* (*A. thaliana*) embryos during seed germination and development demonstrates that GA3-oxidase plays a key regulatory role in controlling the appropriate levels of bioactive GAs during plant growth [7], [8], [9], [10]. GA3-oxidase is able to catalyze the final step of GAs metabolism to produce the bioactive GAs by converting GA_9_ and GA_20_ into bioactive GA_4_ and GA_1_, respectively (Figure 1) [11]. In some species, GA_9_ and GA_20_ are also converted to GA_7_ and GA_3_, respectively, *via* 2,3-didehydroGA_9_ and GA_5_, probably as side reactions of GA3-oxidase [12], [13]. Although the biosynthetic pathway of GAs catalyzed by GA3-oxidase is established and the genes encoding GA3-oxidase have been isolated from *A. thaliana*, the detection of catalytic activity of endogenous GA3-oxidase remains challenging and no method has been reported for the evaluation of endogenous GA3-oxidase so far.

Mass spectrometry (MS)-based methods have been developed for the analysis of some enzymes activities [14], [15], [16], [17]. The advantage of MS-based enzyme assays has the capacity for simultaneously monitoring the multiple pathways catalyzed by the same enzyme. Gas chromatography-MS (GC-MS) method has been employed for the assessment of *in-vitro* recombinant GA3-oxidase activity expressed from *Escherichia coli* cells [18], [19], [20]. But the measurement of endogenous activity of GA3-oxidase in plant hasn't been reported, which probably is due to the lack of highly sensitive method to analyze the substrates and products of limited endogenous GA3-oxidase. Therefore, researchers normally examined the gene expression levels of the GAs oxidases to estimate the endogenous amount of GAs oxidases [21], [22], [23], [24]. Liquid chromatography-MS (LC-MS) method has been employed to analyze GAs [21], [25], [26], however, determination of GAs with particulate-packed columns of 2.1 mm internal diameter (*i.d.*) normally requires relatively large amounts of GAs, which may not be sufficient for the analysis of endogenous GA3-oxidase activity in plant samples.

Capillary LC-MS (*c*LC-MS) has been widely used for the ultra-sensitive analysis of small molecules [27]. By sensitively analyzing the enzymatic substrates and products, *c*LC-MS may service as a promising method for the accurate assay of endogenous GA3-oxidase activity in small amount plant samples. The porous polymer monolithic columns have several unique properties, such as good pH stability, ease of preparation, and numerous commercial monomers, which can provide good separation resolution towards target analytes with mixed-mode chromatographic retention under isocratic elution condition [28], [29], [30]. Recently, we prepared a cation exchange/reversed-phase (CX/RP) mixed mode polymer monolithic column and successfully profiled the acidic phytohormones [31], which suggests that evaluation of endogenous GA3-oxidase may be achieved with *c*LC-MS by sensitively analyzing its substrates and products.

Herein, we developed a highly sensitive and simple method for the determination of endogenous GA3-oxidase activity using a poly([2-(methacryloyloxy)ethyl]trimethylammonium-*co*-divinylbenzene-*co*-ethylene glycol dimethacrylate)(META-*co*-DVB-*co*-EDMA) monolithic column coupled with mass spectrometry. With the existence of quaternary ammonium and phenyl groups on the monolithic surface, five GAs (GA_1_, GA_4_, GA_9_, GA_20_ and GA_53_) were baseline separated by anion exchange/reversed-phase (AX/RP) mixed-mode retention. The in-source collision activated dissociation (CAD) of GAs was suppressed in ESI source by optimizing the ESI conditions. Additionally, a large injection volume (1200 nL) was employed to improve the detection sensitivity. Under optimized conditions, LODs for target GAs range from 0.62 to 0.90 fmol, which is, to the best of our knowledge, the lowest LODs of GAs ever reported without derivatization. Kinetic parameters (*K*_m_) of the recombinant GA3-oxidase were determined to be 1.7 µM and 14.0 µM for GA_9_ and GA_20_, respectively. The developed method was further applied in the evaluation of endogenous GA3-oxidase activity in different types of plant tissues, including rice embryos, rice seedlings, *A. thaliana* seedlings, and *A. thaliana* flowers based on the quantification of isotope labeled catalytic products ([^2^H_2_]GA_1_ and [^2^H_2_]GA_4_) of GA3-oxidase. The results show that endogenous GA3-oxidase activity can be distinctly determined in all the four types of plant tissues examined.

## Materials and Methods

### Chemicals

META (80 wt % in H_2_O, containing 600 ppm monomethyl ether hydroquinone as the inhibitor), DVB (80 wt %, 1000 ppm *p*-tert-butylcatechol as the inhibitor), and EDMA (98 wt % pure, containing 90–110 ppm MEHQ as inhibitor) were purchased from Acros (New Jersey, USA). To remove inhibitors, DVB and EDMA were extracted with 10% aqueous sodium hydroxide and water; after drying with MgSO_4_, they were filtered and distilled under reduced pressure. META was directly used without further purification. Azobisisobutyronitrile (AIBN) and PEG-6000 were purchased from Shanghai Chemical Reagent Corporation (Shanghai, China). AIBN was purified by recrystallization from ethanol at 40°C. 3-(Triethoxysilyl) propyl methacrylate was purchased from Wuhan University Silicone New Material (Wuhan, China). HPLC-grade methanol and acetonitrile (ACN) were obtained from TEDIA (Ohio, USA). The water used throughout all experiments was purified using a Milli-Q water purification system (Millipore, Bradford, USA). The fused-silica capillaries with were purchased from Yongnian Optic Fiber Plant (Hebei, China).

Stable isotope-labeled compounds and standards, [^2^H_2_]GA_1_, [^2^H_2_]GA_4_, [^2^H_2_]GA_9_, [^2^H_2_]GA_20_, [^2^H_2_]GA_53_, GA_1_, GA_4_, GA_9_, and GA_20_ were all purchased from Olchemim Ltd. (Olomouc, Czech Republic). Thiourea, acrylamide, formic acid (FA), *N,N*-dimethylformamide (DMF), ammonium formate (HCOONH_4_), DL-Dithiothreitol (DTT), phenylmethanesulfonyl fluoride (PMSF), tris(hydroxymethyl)aminomethane, NaCl, NaF, Na_3_VO_4_, and other chemicals of analytical grade used in the experiment were purchased from Shanghai Chemical (Shanghai, China). The standard solution (100 µg/mL) for each analyte was prepared in methanol and stored at −4°C in the dark.

### Preparation and Characterization of Poly(META-*co*-DVB-*co*-EDMA) Monolith

The poly(META-*co*-DVB-*co*-EDMA) monolith was prepared by one-step thermally initiated *in-situ* polymerization. Firstly, porogen PEG-6000 was dissolved in DMF and thoroughly mixed to ensure that the solution was completely homogeneous. Subsequently, META, DVB and EDMA were added in the polymerization mixture. The mixture was then briefly shaken before adding the initiator AIBN (1 wt % of monomers). The polymerization mixture was completely mixed by vortex and ultrasonication to form a homogeneous solution after adding AIBN. Then the resulting solution was filled into the capillary. Both ends of the capillary were sealed by silicon rubber for polymerization at 60°C for 12 h. Finally, the prepared monolithic column was washed with ACN to remove residual reagents, and conditioned by the mobile phase (ACN/H_2_O/FA, 60/40/0.6, v/v/v) at 1 µL/min for 1 h.

Poly(META-*co*-DVB-*co*-EDMA) monoliths were also synthesized in Eppendorf vial for the specific surface area measurement. After polymerization, the monolith was cut into small cubic pieces and submersed in ACN/FA (90/10, v/v) for at least 2 h at 60°C to remove the PEG-6000 and non-reacted chemicals. The washing step was repeated three times followed by drying in oven at 80°C. And the resulting monolithic cubic pieces were kept in a desiccator before characterization.

The specific surface area and mesopore size distribution of the prepared poly(META-*co*-DVB-*co*-EDMA) monoliths were measured by nitrogen adsorption-desorption experiments using a JW-BK specific surface area and pore size analyzer (JWGB Sci & Tech Co., Ltd., Beijing, China). Before measurement, the monolithic cubic pieces were evacuated in vacuum and heated to 120°C for 3 h to remove the physically adsorbed substances. Specific surface area values were determined by the Brunauer-Emmett-Teller (BET) equation at P/P_0_ between 0.05 and 0.35 [32]. Mesopore size distributions were determined from the desorption branches of isotherms based on the Barrett-Joyner-Halenda (BJH) model [33].

The microscopic morphology of the monolith was examined by scanning electron microscopy (SEM) using a Quanta 200 scanning electron microscope (FEI Company, Holland). Permeability measurements were performed using a Shimadzu LC-10AT pump (Kyoto, Japan) under the constant flow mode. ACN was pumped through the prepared monolithic column (30 cm-long, 100 µm *i.d.*, 360 µm *o.d.*) at flow rate of 2 µL/min. The back pressure was recorded when the pressure stabilized. Permeability (*K*) was calculated according to Darcy's Law [34].

### Expression of GA3-oxidase in *E. coli* Cells

The coding sequences of *Arabidopsis GA3ox1* were cloned into vector pGEX containing Glutathione S-transferase (GST) tag. The *E. coli* cell lysate expressing GST-GA3ox1 was produced according to previously described protocol [8].

### Preparation of Plant Samples for Evaluation of Endogenous GA3-oxidase Activity

The plant samples (rice and *A. thaliana*) were grown in a growth chamber in a 16 h light/8 h dark photoperiod with 80% humidity under 28°C. Light intensity was fixed to 120 lux/m^2^/s.

All plant samples were collected, weighted, immediately frozen in liquid nitrogen, and then stored at −80°C. Plant samples (2–250 mg) were frozen in liquid nitrogen and finely ground followed by extraction with 8 mL/g cell lysis buffer (25 mM Tris-HCl, pH 7.5, 150 mM NaCl, 2 mM DTT, 1 mM NaF, 0.5 mM Na_3_VO_4_, 15 mM *β*-glycerophosphate, 0.5 mM PMSF) for 30 min, then centrifuged at 10,000 g under 4°C for 5 min to collect the cell lysates. The cell lysates were concentrated to 100 µL at 4°C using ultrafiltration membrane (30 kDa cutoff; Millipore) for the analysis of endogenous GA3-oxidase activity.

### Assessing GA3-oxidase Activity with *c*LC-MS

For enzyme assay, the *E. coli* cell lysates or plant cell lysates (50 µL) were incubated with the substrates (GA_9_ and GA_20_ for *E. coli* cell lysates, [^2^H_2_]GA_9_ and [^2^H_2_]GA_20_ for plant cell lysates) and the internal standard ([^2^H_2_]GA_53_) in the presence of 100 mM Tris-HCl (pH 7.5) and cofactor mixture (4 mM 2-oxoglutarate, 5 mM *L*-ascorbate and 5 mM FeSO_4_) in a total volume of 100 µL. The *E. coli* cell lysate without GA3-oxidase expression was used as negative control. The incubation was performed at 30°C for 3 h and then 150 µL of acetic acid was added to stop the reaction. The resulting solution was lyophilized to dryness and then redissolved in 510 µL H_2_O/ACN (10/500, v/v). The mixture was vortexed and centrifuged at 12,000 g under 4°C for 5 min. The supernatant was collected and evaporated under mild nitrogen stream at 25°C followed by reconstituting in 100 µL H_2_O/ACN (90/10, v/v) for *c*LC-MS analysis.

Determination of the substrates and products of GA3-oxidase was performed on a Shimadzu Prominence nano-flow liquid chromatography system (Kyoto, Japan) coupled with a Bruker Daltonics micrOTOFq orthogonal-accelerated time-of-flight mass spectrometer (Bremen, Germany). The Shimadzu *c*LC system contains two LC-20AD nano pumps, two vacuum degassers, a LC-20AB HPLC pump, a SIL-20AC HT autosampler and a FCV nano valve. The Bruker mass spectrometer is controlled by Bruker Daltonics Control 3.4. Bruker Daltonics Data analysis 3.4 software was employed for the data analysis. Transfer parameters were optimized by direct infusion of an ESI tuning mix from Agilent Technologies (Waldbronn, Germany). Spectra were collected with a time resolution of 50 Hz in the *m/z* range of 50–600.

The analytical column of poly(META-*co*-DVB-*co*-EDMA) monolithic column (100 µm *i.d.*, 360 µm *o.d.*, 30-cm long) was connected to nano-flow liquid chromatographyand conditioned with the mobile phase at a flow rate of 800 nL/min for 30 min. The poly(META-*co*-DVB-*co*-EDMA) monolithic column was coupled with a ESI emitter (7 cm×25 µm, with a 8±1 µm tip) (PicoTip company, USA) by a stainless steel union. The fused-silica capillaries (50 µm *i.d.*) with different lengths were employed as the 800∼1400 nL sample loop (e.g. the volume of 60-cm long capillary was 1200 nL).

## Results and Discussion

### Preparation of poly(META-*co*-DVB-*co*-EDMA) monolithic column

Using binary crosslinkers, DVB and EDMA, a CX/RP polymer monolith with a large specific surface area was successfully prepared in our previous work [35]. DVB can enhance the crosslinking degree of monolith [36], [37], and EDMA can promote to form a homogeneous polymerization mixture when adding hydrophilic/charged functional monomers. Thus, a monolithic column with large specific surface area can be obtained with facile preparation process. In current study, the GA3-oxidase catalytic substrates and products are organic molecules with carboxyl groups; therefore, the positively charged functional monomer META and hydrophobic crosslinkers DVB and EDMA are empolyed to provide a anion-exchnage/RP (AX/RP) mixed-mode chromatographic retention towards analytes.

To obtain the favourable porous structure of the poly(META-*co*-DVB-*co*-EDMA) monolith, the polymerization mixture was optimized (Table S1, S2, S3, Figure S1). The optimized polymerization mixture consists of 3.7% (w/w_total_) META, 11.0% (w/w_total_) DVB, 11.0% (w/w_total_) EDMA, 11.4% (w/w_total_) PEG-6000, 62.9% (w/w_total_) DMF and 1% (w/w_total monomers_) AIBN.

### Characterization of poly(META-*co*-DVB-*co*-EDMA) Monolithic Column

**Figure 2:**
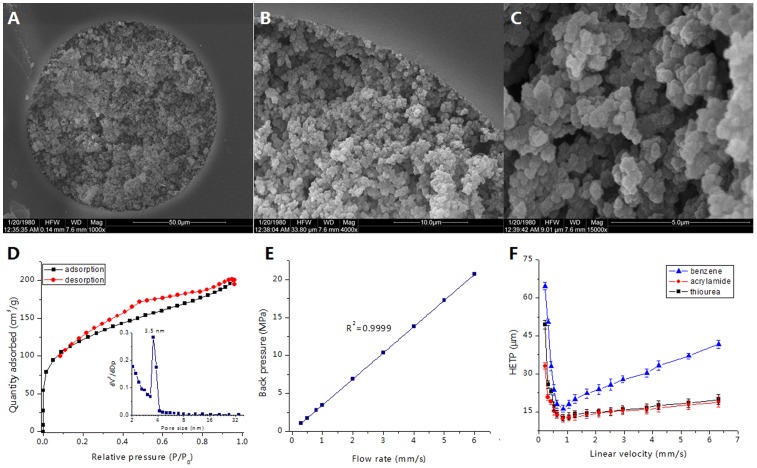
Characterizations of the META-silica hybrid monolithic column. (A) – (C) SEM images. (A) ×1,000 wide-view, (B) ×4,000 close-up-view, and (C) ×15,000 close-up-view. (D) The N_2_ isothermal plot with the inset showing the pore-size distribution. (E) The effect of flow rate on the back pressure of the monolithic column. (F) Van Deemter plot of the height equivalent to a theoretical plate as a function of flow rate. Experimental conditions: column, poly(META-*co*-DVB-*co*-EDMA) monolithic column (30-cm long, 100 µm *i.d.*, 360 µm *o.d.*); UV detection wavelength, 254 nm for acrylamide, 214 nm for thiourea and benzene; mobile phase used in (E), ACN; mobile phase used in (F), ACN/H_2_O (60/40, v/v).

The morphology of the prepared poly(META-*co*-DVB-*co*-EDMA) monolithic column was examined by SEM (Figure 2A–C). Figure 2A shows the coarse surface of microglobules, which indicates the existence of mesopores on the monolithic surface. In addition, the continuous monolithic matrix was obtained and attached well to the inner wall of the monolithic column (Figure 2B) with through-pores of approximate 1.5 µm (Figure 2C). The distribution of mesopore in the poly(META-*co*-DVB-*co*-EDMA) monolith ranges from 3.3 to 4.0 nm, and the specific surface area is 426 m^2^/g (Figure 2D), which is comparable to the hypercrosslinked monolithic column [38], [39], [40].

The mechanical stability of the prepared monolithic column was examined with an increase of flow rate from 100 to 1800 nL/min (linear velocity: 0.21–5.88 mm/s). The results show that the back pressure linearly increases with the increase of the flow rate (Figure 2E), which demonstrates that the monolithic column bed is stable even under high back pressure (∼20 MPa).

The column efficiency of the poly(META-*co*-DVB-*co*-EDMA) monolith was evaluated by changing the flow rate from 0.05 to 2.50 mm/s. The effect of the flow velocity on the plate height was examined by using benzene, acrylamide, and thiourea (Figure 2F). The results show the lowest plate height is approximate 12.2 µm for acrylamide with a flow velocity of 0.84 mm/s (∼82,000 plates/m). Moreover, under high velocity, the theoretical plate heights of these analytes did not significantly increase, which suggests fast separation of GAs can be implemented with this monolithic column.

The reproducibility of the prepared poly(META-*co*-DVB-*co*-EDMA) monoliths was evaluated by analysis of GA_1_, benzene and acrylamide. The run-to-run (n = 10), column-to-column (n = 5) and batch-to-batch (n = 5) variations (RSDs) of these monoliths were 0.9%, 5.7%, and 11.4%, respectively, for retention time of these analytes, and 1.4%, 8.8%, and 13.7%, respectively, for the column efficiency of these three analytes. Additionally, the retention time and column efficiency of analytes and the back pressure of monolithic column did not significantly change even after three-month continuous use. These results indicate that excellent reproducibility of the monoliths can be achieved.

### Separation of Target GAs on Poly(META-*co*-DVB-*co*-EDMA) Monolithic Column

We investigated the separation mechanisms and optimized the separation conditions for five GAs (GA_1_, GA_4_, GA_9_, GA_20_, and GA_53_) with *c*LC-MS. Since the existent of salt in the mobile phase can lower the MS sensitivity of GAs, buffer solution was not used in the mobile phase for the subsequent experiment.

**Figure 3:**
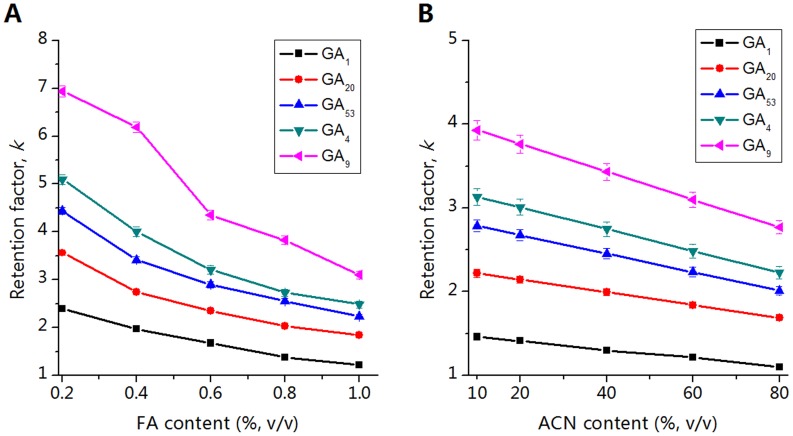
Investigation of the separation mechanisms for five GAs. (A) The effects of FA content on the retention factor (*k*) of GAs. (B) The effects of ACN content on the retention factor (*k*) of GAs. Experimental conditions: column, poly(META-*co*-DVB-*co*-EDMA) monolithic column (30-cm long, 100 µm *i.d.*, 360 µm *o.d.*).

Firstly, the influence of mobile phase acidity on the retention of GAs was investigated. Without a buffer solution, the addition of formic acid (FA) will lead to the immediate decrease of the mobile phase pH to approximately 3.5, and further increase of FA content could affect the mobile phase pH slightly. Whereas, the ion strength of the mobile phase will increase by adding FA. Thus, we optimized the FA content in the mobile phase instead of the mobile phase pH. In Figure 3A, when the FA content increased from 0.2 to 1% (v/v), the retention factors (*k*) of GAs decrease. We reason that with the increase of FA content, the degree of ionization of GAs is suppressed and the ion strength of mobile phase increased, therefore, the electrostatic interaction between GAs and the quaternary ammonium groups is weaken, which exhibits typical anion-exchange retention mechanism towards GAs. The increase of ACN content in mobile phase from 10 to 80% (v/v) results in the linear decrease of the retention of GAs (Figure 3B), which indicates the RP retention of the monolithic column. Taken together, the results demonstrate the SAX/RP retention mechanism of poly(META-*co*-DVB-*co*-EDMA) monolithic column towards negatively charged GAs. Considering the high ACN content can improve the MS response of GAs, ACN content was kept at 60% (v/v) for the subsequent experiment.

**Figure 4:**
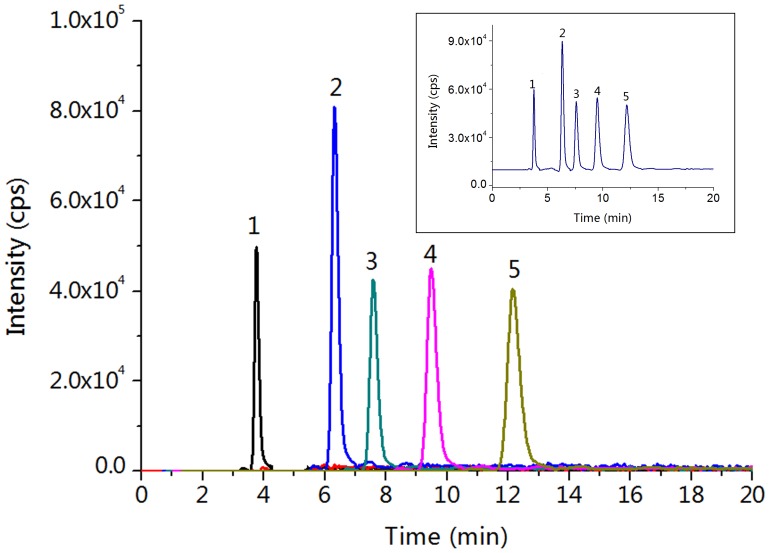
Extracted ion chromatogram of five GA standards. Shown in inset is the total ion chromatogram of five GA standards. Experimental conditions: column, poly(META-*co*-DVB-*co*-EDMA) monolithic column (30-cm long, 100 µm *i.d.*, 360 µm *o.d.*); flow rate, 800 nL/min; mobile phase, ACN/H_2_O/FA (60/40/0.6, v/v/v). Order of peaks: 1. GA_1_, 2. GA_20_, 3. GA_53_, 4. GA_4_, 5. GA_9_.

In addition, the separation resolution of these analytes was not apparently influenced with a high flow rate (Figure S2). At the flow rate of 500 nL/min, the column efficiencies of 5 GAs are around 75,000 plates/m. With the increase of flow rate, the column efficiencies of all five GAs slightly decreased from 75,000 to 60,000 plates/m, which indicated that this polymer monolithic column with large specific surface area is suitable for the fast separation of acidic small molecules. Yet, the nano-valves and tubes were frequently blocked after sample injection when the back pressure exceeding 10 MPa. Thus, we chose 800 nL/min as the optimal flow rate for the further methodological investigation. Finally, a 14-min baseline separation of 5 GAs was achieved with this poly(META-*co*-DVB-*co*-EDMA) monolithic column using the isocratic elution condition of ACN/H_2_O/FA (60/40/0.6, v/v/v) at the flow rate of 800 nL/min (Figure 4).

### Optimization of ESI-MS Conditions for Detection of GAs

**Figure 5:**
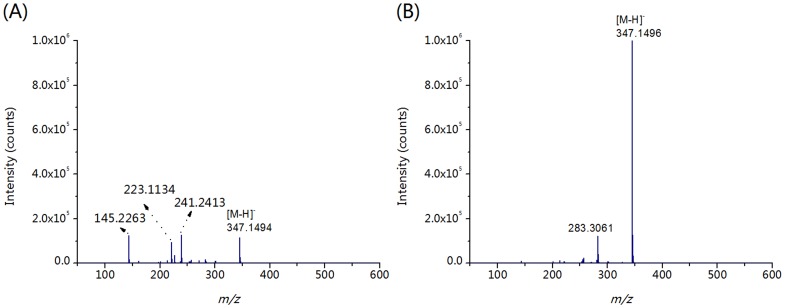
Optimization of the mass spectrometry detection conditions of GA_1_. (A) Full-scan spectrum of GA_1_. (B) Full-scan spectrum of GA_1_ with optimized ESI source conditions. Experimental conditions: 1 µg/mL GA_1_ were infused in mobile phase (ACN/H_2_O/FA, 60/40/0.6, v/v/v) at a flow rate of 3 µL/min.

In previous reports, the acidic phytohormones were frequently analyzed in multiple reaction monitoring (MRM) mode. Whereas, the fragmentation behaviour of GAs hasn't been rigorously investigated. According to the full-scan spectrum (ESI-MS spectrum) of GA_1_ (Figure 5A), the [M - H]^−^ adduct of GA_1_ appeared at *m/z* 347.1494. Also present, however, was the in-source CAD fragment ion at *m/z* 145.2263, 223.1134, and 241.2413. The intensity of fragment ions at *m*/*z* 145.2263 (*I*_145_), 223.1134 (*I*_223_), and 241.2413 (*I*_241_) versus the intensity of GA_1_ at *m*/*z* 347.1494 (*I*_347_) was 1.2/1, 0.9/1, and 1/1, respectively. These observations indicate that GAs was a set of fragile molecules and the in-source CAD occurrence can cause the loss in the detection sensitivity of GAs.

To circumvent this problem, we optimized the in-source ESI-MS conditions to suppress the in-source CAD occurrence of GAs, including funnel radio frequency (RF), collision RF, hexapole RF, capillary voltage, flow rate and temperature of dry gas. The results show that, under optimized in-source ESI-MS conditions (see details in Supporting Information), the *I*_145_, *I*_223_, and *I*_241_ versus *I*_345_ are all lower than 1/10, which increases the detection sensitivity of GA_3_ for more than one order of magnitude (Figure 5B). Consequently, the ion of *I*_347_ (347.1496) was used for the quantification of GA_1_. The ESI source conditions for the analysis of other 4 GAs were also optimized using the same strategy (Figure S3).

### Methodological Establishment

Since the prepared poly(META-*co*-DVB-*co*-EDMA) monolithic column possesses large specific surface area of 426 m^2^/g and fast mass transfer kinetics, a large sample injection volume can be implemented on this monolithic column with no decreased separation resolution. The target GAs can be trapped in the front of analytical monolithic column with aqueous sample matrix. Subsequently, the mobile phase with high ACN can elute GAs from the sample zone. The signal/noise (S/N) ratio for most of the GAs increases with the increase of injection volume from 800 nL to 1200 nL, while the injection volume of 1400 nL results in a decrease of S/N ratio (Table S4), which indicates the diffusion of sample zone on the monolithic column. Consequently, sample injection volume was fixed at 1200 nL for further experiments.

The LODs and LOQs of GAs are calculated as the amounts of the analyte at an S/N ratio of 3 and 10, respectively. The results show the LOD and LOQ were 0.62 and 2.00 fmol for GA_1_, and 0.90 and 3.04 fmol for GA_4_, respectively (Table 1), which was comparable to that obtained by derivatization method [31], [41]. The detection linearity of the method was investigated using the internal standard of 30.0 fmol [^2^H_2_]GA_53_ spiked with GA_1_ and GA_4_ at different amounts ranging from 2.00 fmol to 400 fmol. The calibration curve was constructed by plotting the mean peak area ratio of GA_1_ and GA_4_ to [^2^H_2_]GA_53_ versus their molar amount based on data obtained from triplicate measurements. The results show that good linearity within the range of 2.00–400 fmol GAs was obtained with coefficient correlation (R) higher than 0.9971 (Table 1).

**Table 1:** Linearity, LODs and LOQs of GAs obtained by *c*LC-MS method.

Analytes	Linear range (fmol)	Regression line		R	LOD (fmol)	LOQ (fmol)
		Slope	Intercept			
GA_1_	2.00–400	0.0025	−0.0733	0.9971	0.62	2.00
GA_4_	2.00–400	0.0041	−0.0961	0.9975	0.90	3.04

We then examined the stabilities of the GA3-oxidase catalytic products ([^2^H_2_]GA_1_ and [^2^H_2_]GA_4_) and internal standard ([^2^H_2_]GA_53_) under the enzyme catalytic reaction conditions. The results show that there were no apparent changes of the MS intensities for these analytes in the reaction mixture even for 24 h, which demonstrates that the method is appropriate for the evaluation of endogenous GA3-oxidase activity by measuring GA3-oxidase catalytic products of [^2^H_2_]GA_1_ and [^2^H_2_]GA_4_.

The effect of matrix on the quantification of these bioactive GAs was evaluated by spiking standards (2.00–400 fmol GA_1_, GA_4_, and 30 fmol [^2^H_2_]GA_53_) into *E. coli* cell lysates or isotope standards (2.00–400 fmol [^2^H_2_]GA_1_, [^2^H_2_]GA_4_, and 30 fmol [^2^H_2_]GA_53_) into rice seedling matrix. The results show that these GAs can be successfully determined with 89.5–116.0% recoveries (RSDs, 1.8–11.9%, N = 4) in *E. coli* cell lysates (Table S5) and 80.2–93.2% recoveries in rice seedling matrix (RSDs, 0.2–7.8%, N = 4) (Table S6). Additionally, good intra- and inter-day precision can be achieved, which were manifested by RSDs (N = 5) being less than 11.2% and 13.5% with *E. coli* cell lysates (Table S7) and less than 9.3% and 10.7% with rice seedling matrix (Table S8). Taken together, these results indicate that the *c*LC-MS method is reliable for the quantification of GA3-oxidase catalytic products.

### Measurement of GA3-oxidase Activity by *c*LC-MS

**Figure 6:**
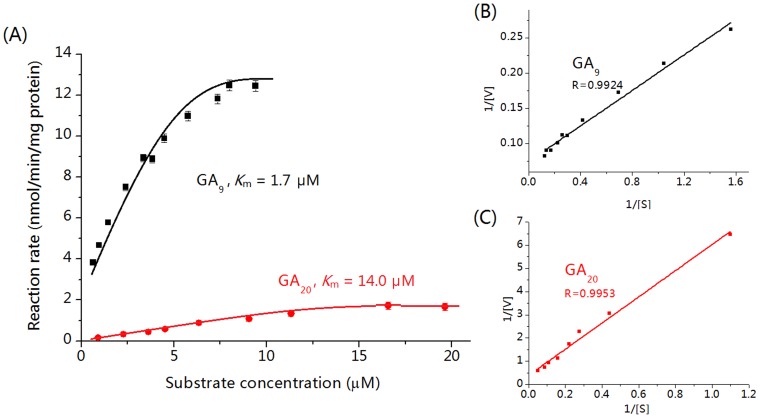
The kinetics study of GA3-oxidase. (A) Michaelis–Menten plots of recombinant GA3-oxidase in *E. coli*. cell lysate. (B) Lineweaver–Burk plots for GA_9_. (C) Lineweaver–Burk plots for GA_20_. Experimental conditions: GA_9_ or GA_20_ was incubated for 15 min at 30°C with *E. coli*. cell lysate and cofactors.

The kinetic parameters (*K*_m_) of *in-vitro* recombinant GA3-oxidase determined by our developed *c*LC-MS method was 1.7 µM and 14.0 µM for GA_9_ and GA_20_, respectively (Figure 6), which are consistent with previous reports (1.0–1.5 µM for GA_9_, and 13.0–15.0 µM for GA_20_) [13], [14], [15], [16]. Additionally, the much lower *K*_m_ value of GA3-oxidase for GA_9_ than GA_20_ suggests the higher catalytic activity of GA3-oxidase towards GA_9_ than GA_20_.

**Figure 7:**
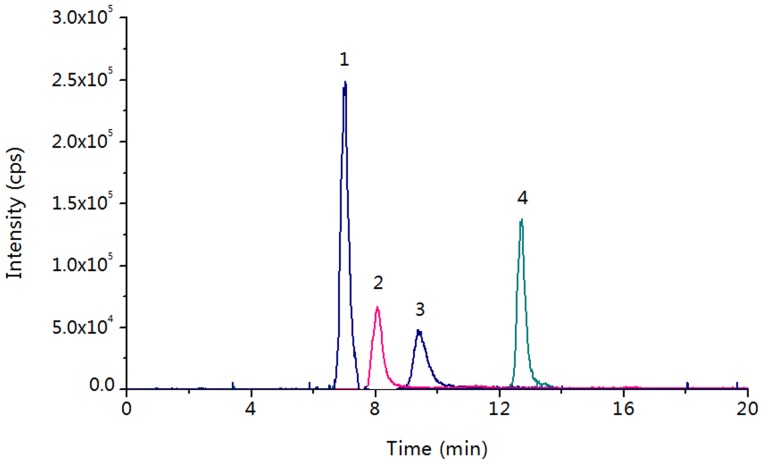
Extracted ion chromatogram of the catalytic products and substrates of GA3-oxidase using 5 mg of rice embryos. Experimental conditions: column, poly(META-*co*-DVB-*co*-EDMA) monolithic column (30-cm long, 100 µm *i.d.*, 360 µm *o.d.*); flow rate, 800 nL/min; mobile phase, ACN/H_2_O/FA (60/40/0.6, v/v/v). Order of peaks: 1. [^2^H_2_]GA_20_, 2. [^2^H_2_]GA_53_ (I.S.), 3. [^2^H_2_]GA_4_, 4. [^2^H_2_]GA_9_.

According to previous reports, the endogenous bioactive GAs could be produced by GA3-oxidase catalysis in plant germinating, seedling and flowering [7], [8], [9], [10], [42]. However, up to date, the quantification of endogenous GA3-oxidase activity in plant species has not been established. With our developed *c*LC-MS method, we determined the catalytic activity of endogenous GA3-oxidase for converting GA_9_ to GA_4_ in rice embryos (1.01±0.24 pmol/g/h, n = 3), rice seedlings (0.21±0.03 pmol/g/h, n = 3), *A. thaliana* seedlings (1.77±0.36 pmol/g/h, n = 3), and *A. thaliana* flowers (0.22±0.04 pmol/g/h, n = 3). The extracted ion chromatogram of enzyme catalytic products [^2^H_2_]GA_4_ in rice embryos exhibited that the products can be clearly identified and quantified with no interfere (Figure 7). [^2^H_2_]GA_1_, which was another GA3-oxidase catalytic product by converting [^2^H_2_]GA_20_, was not observed even using 1 g plant sample. This result suggests that endogenous GA3-oxidase has much lower catalytic activity towards GA_20_ than GA_9_, which is consistent with the results obtained using *in-vitro* recombinant GA3-oxidase (the *K*_m_ of recombinant GA3-oxidase for GA_20_ was approximate one order of magnitude larger than that for GA_9_).

In addition, we investigated the minimal plant sample required for the quantification of endogenous GA3-oxidase activity. With the decrease of sample amount from 250 to 5 mg, the endogenous GA3-oxidase catalytic activity (pmol/h) linearly decreased (Figure S4). The activity of endogenous GA3-oxidase can be distinctly measured from 5 mg plant samples. Compared to the normal LC-MS method, our developed *c*LC-MS method can achieve much better detection sensitivity towards GAs [43], therefore, the low activity of endogenous GA3-oxidase can be easily assessed from small amount plant sample by analyzing its substrates and products.

## Conclusions

In this study, we developed a sensitive and robust method for the evaluation of recombinant or endogenous GA3-oxidase activity using poly(META-*co*-DVB-*co*-EDMA) monolithic column coupled with mass spectrometry. With AX/RP mixed-mode chromatographic retention mechanism, the catalytic substrates and products of GA3-oxidase (GA_1_, GA_4_, GA_9_, GA_20_) and internal standard of [^2^H_2_]GA_53_ were well separated. Using this method, we investigated the *K*_m_ of recombinant GA3-oxidase, which was consistent with previous reports. Additionally, the activity of endogenous GA3-oxidase catalyzing the conversion of [^2^H_2_]GA_9_ into [^2^H_2_]GA_4_ was successfully determined in four different types of plant samples. These results demonstrate the endogenous GA3-oxidase activity can be distinctly measured by our developed *c*LC-MS method, which provides appropriate analytical tool for the further study of the growth regulation mechanism controlled by GAs.

## Supporting Information

Text S1 Optimization for the Preparation of Poly(META-*co*-DVB-*co*-EDMA) Monolithic Column.
(DOC)

Text S2 Optimization of ESI-MS Conditions for Detection of GAs.
(DOC)

Table S1 Permeability (*K*) and microscopic images of the monoliths prepared with different amount of PEG-6000.
(DOC)

Table S2 Optimization of the amount of META for the preparation of monoliths.
(DOC)

Table S3 Optimization of the weight of DVB to EDMA for the preparation of monoliths.
(DOC)

Table S4 The signal/noise (S/N) ratios of 4 target GAs with different sample injection volume.
(DOC)

Table S5 Recoveries for the determination of GA3-oxidase catalytic products (GA_1_, and GA_4_) in *E. coli* cell lysate.
(DOC)

Table S6 Recoveries for the determination of GA3-oxidase catalytic products ([^2^H_2_]GA_1_ and [^2^H_2_]GA_4_) in rice seedling sample.
(DOC)

Table S7 Precisions (intra- and inter-day) for the determination of GA3-oxidase catalytic products (GA_1_ and GA_4_) in the matrix of *E. coli* cell lysate.
(DOC)

Table S8 Precisions (intra- and inter-day) for the determination of GA3-oxidase catalytic products ([^2^H_2_]GA_1_ and [^2^H_2_]GA_4_) in rice seedling sample.
(DOC)

Figure S1 The Scanning electron microscope images of the cross section of monoliths (×16,000 close-up-view).
(DOC)

Figure S2 Extracted ion chromatograms of 5 GAs with different linear velocity.
(DOC)

Figure S3 Full-scan spectra of GAs under optimized ESI source conditions.
(DOC)

Figure S4 The linearity of endogenous GA3-oxidase activity (pmol/h) with different plant weight (mg).
(DOC)
