# Supplementary material for: Assessing Gibberellins Oxidase Activity by Anion Exchange/Hydrophobic Polymer Monolithic Capillary Liquid Chromatography-Mass Spectrometry
Source: PLoS One. 2013 Jul 26;8(7):e69629. doi: 10.1371/journal.pone.0069629 (PMC3724942; doi:10.1371/journal.pone.0069629)
Supplement: Text S1 — Optimization for the Preparation of Poly(META-co-DVB-co-EDMA) Monolithic Column. (DOC) [file pone.0069629.s001.doc]

**Text S1: Optimization for the Preparation of Poly(META-*co*-DVB-*co*-EDMA) Monolithic Column**

Since poly(ethylene glycol) with the molecular weight of 6000 (PEG-6000) can influence the relative rate of phase separation to polymerization and the porous structure, we firstly evaluated the effect of porogen PEG-6000 content from 6.3 to 16.7 % (w/w_total_) on the permeability and homogeneity of the monolithic columns (column 1 – 5, Table S1). The results show that homogeneous columns can be obtained using 11.8 % (w/w_total_) of PEG-6000 with appropriate permeability of 7.4 × 10^-14^ m^2^.

Then, the weight ratio of functional monomers META to total monomers was optimized by changing their ratio from 1/9 to 2/7 (w/w_total monomers_) (column 6 – 9, Table S2). We found that homogeneous monoliths can be obtained when the weight ratios of META to crosslinkers were 1/9, 1.5/9 and 2/9 (w/w_total monomers_) (column 6 – 8, Table S2). The monoliths became slack when the ratio increased to 2/7 (w/w_total monomers_) (column 9, Table S2). We reason that the low content of crosslinkers was not sufficient to form stable bond with the inner wall of capillary. Column 6 showes the low permeability of 0.7 × 10^-14^ m^2^, which was not suitable for chromatographic applications. Column 7 has the smaller size of microglobules and larger specific surface than column 8 (Fig. S1). Therefore, the weight ratio of META to total monomers of 1.5/9 (column 7, Table S2) was employed for further experiments.

Furthermore, the weight ratio of DVB to EDMA was optimized from 3/4 to 9/5 (column 10, 7, 11 and 12, Table S3). The results show that with the increased ratio of DVB to EDMA from 3/4 to 1/1 (w/w), the permeability decreased from 26.3×10^-14^ to 5.2×10^-14^ m^2^, and the specific surface area of resulting monoliths increased from 203 ± 10 to 426 ± 12 m^2^/g (column 7 and 10, Table S3). While the higher ratio of DVB to EDMA (4/3 and 9/5, w/w) caused the column bed slack though the specific surface area would slightly increased (column 11 and 12, Table S3). Additionally, column 7 with the smaller size of microglobules (Fig. S1) can provide better column efficiency than column 10, thus the weight ratio of DVB to EDMA of 1:1 was employed in the subsequent studies.

Collectively, the optimized polymerization mixture consists of 3.7 % (w/w_total_) META, 11.0 % (w/w_total_) DVB, 11.0 % (w/w_total_) EDMA, 11.4 % (w/w_total_) PEG-6000, 62.9 % (w/w_total_) DMF and 1 % (w/w_total monomers_) AIBN.
