# Supplementary material for: Assessing Gibberellins Oxidase Activity by Anion Exchange/Hydrophobic Polymer Monolithic Capillary Liquid Chromatography-Mass Spectrometry
Source: PLoS One. 2013 Jul 26;8(7):e69629. doi: 10.1371/journal.pone.0069629 (PMC3724942; doi:10.1371/journal.pone.0069629)
Supplement: Text S2 — Optimization of ESI-MS Conditions for Detection of GAs. (DOC) [file pone.0069629.s002.doc]

**Text S2: Optimization of ESI-MS Conditions for Detection of GAs**

To suppress the in-source CAD occurrence of GAs, we optimized the ESI-MS conditions. The optimized ESI-MS conditions are as follows: funnel radio frequency (RF), 250 Vpp; collision RF, 450 Vpp; hexapole RF, 400 Vpp; capillary voltage, 1500 V; flow rate of dry gas, 3.5 L/min; temperature of dry gas, 150ºC.
