# Supplementary material for: Assessing Gibberellins Oxidase Activity by Anion Exchange/Hydrophobic Polymer Monolithic Capillary Liquid Chromatography-Mass Spectrometry
Source: PLoS One. 2013 Jul 26;8(7):e69629. doi: 10.1371/journal.pone.0069629 (PMC3724942; doi:10.1371/journal.pone.0069629)
Supplement: Table S1 — Permeability (K) and microscopic images of the monoliths prepared with different amount of PEG-6000. (DOC) [file pone.0069629.s003.doc]

**Table S1.** Permeability (*K*) and microscopic images of the monoliths prepared with different amount of PEG-6000.^a^

| Column | PEG-6000  (%, w/w_total_) | Status of column | Permeability, *K*  (× 10^-14^ m^2^) | Microscopic  images |
| --- | --- | --- | --- | --- |
| 1 | 6.3 | Gelation | → 0 | - |
| 2 | 9.1 | Gelation | → 0 | - |
| 3 | 11.8 | Homogeneous | 7.4 ± 0.1 | 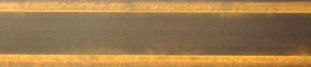 |
| 4 | 14.3 | Slightly Slack | 9.8 ± 0.1 | 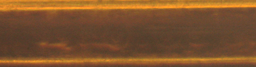 |
| 5 | 16.7 | Slack | 37.2 ± 1.9 | 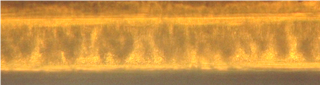 |

^a^ The weight ratio of monomers (META, DVB and EDMA) to DMF was kept at 4/11 (w/w). The ratio of META, DVB and EDMA (w/w_total monomers_) was kept at 1/3/3 (w/w/w).
