# Supplementary material for: Assessing Gibberellins Oxidase Activity by Anion Exchange/Hydrophobic Polymer Monolithic Capillary Liquid Chromatography-Mass Spectrometry
Source: PLoS One. 2013 Jul 26;8(7):e69629. doi: 10.1371/journal.pone.0069629 (PMC3724942; doi:10.1371/journal.pone.0069629)
Supplement: Table S2 — Optimization of the amount of META for the preparation of monoliths. (DOC) [file pone.0069629.s004.doc]

**Table S2. Optimization of the amount of META for the preparation of monoliths.^a^**

| Column | META/total monomers  (w/w) | Status of column | Permeability, *K*  (× 10^-14^ m^2^) | Microscopic  images |
| --- | --- | --- | --- | --- |
| 6 | 1/9 | Homogeneous | 0.7 ± 0.1 | 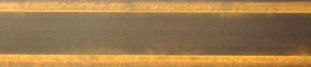 |
| 7 | 1.5/9 | Homogeneous | 5.2 ± 0.1 | 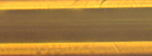 |
| 8 | 2/9 | Homogeneous | 5.7 ± 0.3 | 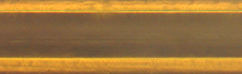 |
| 9 | 2/7 | Slack | 42.9 ± 1.6 | 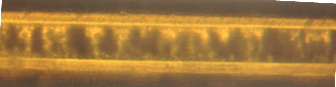 |

^a^ The ratio of monomers (META, DVB and EDMA), PEG-6000 and DMF was kept at 9/4/22 (w/w/w). The ratio of DVB to EDMA was kept at 1/1 (w/w).
