# Supplementary material for: Assessing Gibberellins Oxidase Activity by Anion Exchange/Hydrophobic Polymer Monolithic Capillary Liquid Chromatography-Mass Spectrometry
Source: PLoS One. 2013 Jul 26;8(7):e69629. doi: 10.1371/journal.pone.0069629 (PMC3724942; doi:10.1371/journal.pone.0069629)
Supplement: Table S3 — Optimization of the weight of DVB to EDMA for the preparation of monoliths. (DOC) [file pone.0069629.s005.doc]

**Table S3. Optimization of the weight of DVB to EDMA for the preparation of monoliths.^a^**

| Column | DVB/EDMA  (w/w) | Status of column | Permeability, *K*  (× 10^-14^ m^2^) | Specific surface area  (m^2^/g) | Microscopic  images |
| --- | --- | --- | --- | --- | --- |
| 10 | 3/4 | Homogeneous | 26.3 ± 1.0 | 203 ± 10 | 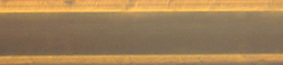 |
| 7 | 1/1 | Homogeneous | 5.2 ± 0.1 | 426 ± 12 | 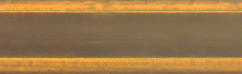 |
| 11 | 4/3 | Slightly slack | 39.6 ± 0.2 | 454 ± 14 | 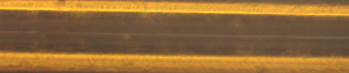 |
| 12 | 9/5 | Slack | 6.4 ± 2.2 | 491 ± 19 | 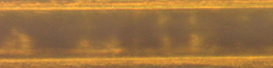 |

^a^ The ratio of monomers (META, DVB and EDMA), PEG-6000 and DMF was kept at 9/4/22 (w/w/w). The ratio of META to DVB&EDMA was kept at 1/6 (w/w).
