# Supplementary material for: Assessing Gibberellins Oxidase Activity by Anion Exchange/Hydrophobic Polymer Monolithic Capillary Liquid Chromatography-Mass Spectrometry
Source: PLoS One. 2013 Jul 26;8(7):e69629. doi: 10.1371/journal.pone.0069629 (PMC3724942; doi:10.1371/journal.pone.0069629)
Supplement: Table S4 — The signal/noise (S/N) ratios of 4 target GAs with different sample injection volume. (DOC) [file pone.0069629.s006.doc]

**Table S4.** The signal/noise (S/N) ratios of 4 target GAs with different sample injection volume.

| Analyte | S/N ratio (*N*=3) | | | |
| --- | --- | --- | --- | --- |
|  | 800 nL | 1000 nL | 1200 nL | 1400 nL |
| GA_1_ | 6823 ± 252 | 8241 ± 395 | 8617 ± 387 | 11752 ± 293 |
| GA_4_ | 6824 ± 996 | 9399 ± 648 | 11981 ± 1210 | 10925 ± 863 |
| GA_9_ | 5868 ± 475 | 8065 ± 580 | 8901 ± 1237 | 10521 ± 831 |
| GA_20_ | 6420 ± 783 | 7841 ± 744 | 9958 ± 1045 | 9635 ± 770 |
