# Supplementary material for: Assessing Gibberellins Oxidase Activity by Anion Exchange/Hydrophobic Polymer Monolithic Capillary Liquid Chromatography-Mass Spectrometry
Source: PLoS One. 2013 Jul 26;8(7):e69629. doi: 10.1371/journal.pone.0069629 (PMC3724942; doi:10.1371/journal.pone.0069629)
Supplement: Table S5 — Recoveries for the determination of GA3-oxidase catalytic products (GA1, and GA4) in E. coli cell lysate. (DOC) [file pone.0069629.s007.doc]

**Table S5.** Recoveries for the determination of GA3-oxidase catalytic products (GA_1_, and GA_4_) in *E. coli* cell lysate.

| Analytes | Recovery (%, *N*=4) | | |
| --- | --- | --- | --- |
|  | Low  (5.00 fmol) | Medium  (50.0 fmol) | High  (300 fmol) |
| GA_1_ | 89.5 ± 10.3 | 103.9 ± 2.6 | 97.1 ± 2.3 |
| GA_4_ | 116.0 ± 10.4 | 106.2 ± 3.2 | 100.7 ± 3.1 |
