# Supplementary material for: Assessing Gibberellins Oxidase Activity by Anion Exchange/Hydrophobic Polymer Monolithic Capillary Liquid Chromatography-Mass Spectrometry
Source: PLoS One. 2013 Jul 26;8(7):e69629. doi: 10.1371/journal.pone.0069629 (PMC3724942; doi:10.1371/journal.pone.0069629)
Supplement: Table S6 — Recoveries for the determination of GA3-oxidase catalytic products ([2H2]GA1 and [2H2]GA4) in rice seedling sample. (DOC) [file pone.0069629.s008.doc]

**Table S6.** Recoveries for the determination of GA3-oxidase catalytic products ([^2^H_2_]GA_1_ and [^2^H_2_]GA_4_) in rice seedling sample.

| Analytes | Recovery (%, *N*=4) | | |
| --- | --- | --- | --- |
|  | Low  (5.00 fmol) | Medium  (50.0 fmol) | High  (300 fmol) |
| [^2^H_2_]GA_1_ | 88.2 ± 0.3 | 93.2 ± 2.0 | 90.0 ± 2.1 |
| [^2^H_2_]GA_4_ | 87.6 ± 5.3 | 91.5 ± 7.2 | 87.2 ± 6.4 |
