# Supplementary material for: Assessing Gibberellins Oxidase Activity by Anion Exchange/Hydrophobic Polymer Monolithic Capillary Liquid Chromatography-Mass Spectrometry
Source: PLoS One. 2013 Jul 26;8(7):e69629. doi: 10.1371/journal.pone.0069629 (PMC3724942; doi:10.1371/journal.pone.0069629)
Supplement: Table S7 — Precisions (intra- and inter-day) for the determination of GA3-oxidase catalytic products (GA1 and GA4) in the matrix of E. coli cell lysate. (DOC) [file pone.0069629.s009.doc]

**Table S7.** Precisions (intra- and inter-day) for the determination of GA3-oxidase catalytic products (GA_1_ and GA_4_) in the matrix of *E. coli* cell lysate.

| Analytes | Intra-day precision (RSD %, *N*=5) | | | Inter-day precision (RSD %, *N*=5) | | |
| --- | --- | --- | --- | --- | --- | --- |
|  | Low  (1 fmol) | Medium  (5 fmol) | High  (10 fmol) | Low  (1 fmol) | Medium  (5 fmol) | High  (10 fmol) |
| GA_1_ | 10.1 | 5.1 | 1.0 | 13.5 | 6.3 | 2.4 |
| GA_4_ | 5.6 | 0.4 | 5.0 | 9.3 | 5.8 | 4.5 |
