# Supplementary material for: Assessing Gibberellins Oxidase Activity by Anion Exchange/Hydrophobic Polymer Monolithic Capillary Liquid Chromatography-Mass Spectrometry
Source: PLoS One. 2013 Jul 26;8(7):e69629. doi: 10.1371/journal.pone.0069629 (PMC3724942; doi:10.1371/journal.pone.0069629)
Supplement: Table S8 — Precisions (intra- and inter-day) for the determination of GA3-oxidase catalytic products ([2H2]GA1 and [2H2]GA4) in rice seedling sample. (DOC) [file pone.0069629.s010.doc]

**Table S8.** Precisions (intra- and inter-day) for the determination of GA3-oxidase catalytic products ([^2^H_2_]GA_1_ and [^2^H_2_]GA_4_) in rice seedling sample.

| Analytes | Intra-day precision (RSD %, *N*=5) | | | Inter-day precision (RSD %, *N*=5) | | |
| --- | --- | --- | --- | --- | --- | --- |
|  | Low  (1 fmol) | Medium  (5 fmol) | High  (10 fmol) | Low  (1 fmol) | Medium  (5 fmol) | High  (10 fmol) |
| [^2^H_2_]GA_1_ | 8.0 | 8.4 | 8.2 | 9.7 | 8.8 | 11.3 |
| [^2^H_2_]GA_4_ | 9.0 | 9.1 | 8.6 | 9.6 | 9.4 | 10.7 |
