# Supplementary material for: Assessing Gibberellins Oxidase Activity by Anion Exchange/Hydrophobic Polymer Monolithic Capillary Liquid Chromatography-Mass Spectrometry
Source: PLoS One. 2013 Jul 26;8(7):e69629. doi: 10.1371/journal.pone.0069629 (PMC3724942; doi:10.1371/journal.pone.0069629)
Supplement: Figure S1 — The Scanning electron microscope images of the cross section of monoliths (×16,000 close-up-view). (DOC) [file pone.0069629.s011.doc]

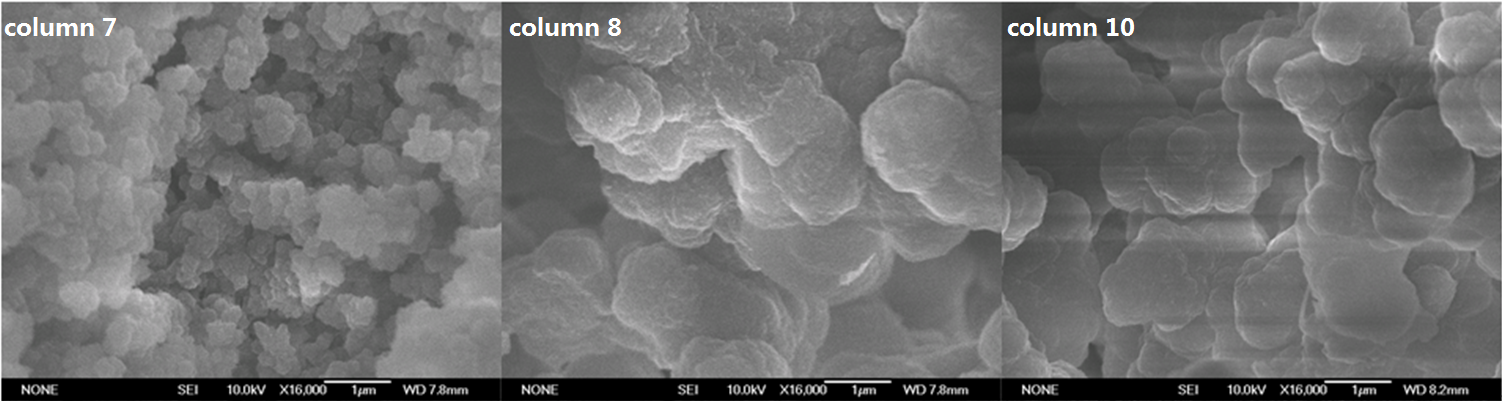


**Figure S1.** The Scanning electron microscope images of the cross section of monoliths (× 16,000 close-up-view). The detailed information of three types of monoliths (column 7, 8, and 10) was provided in Table S2, S3.
