# Supplementary material for: Assessing Gibberellins Oxidase Activity by Anion Exchange/Hydrophobic Polymer Monolithic Capillary Liquid Chromatography-Mass Spectrometry
Source: PLoS One. 2013 Jul 26;8(7):e69629. doi: 10.1371/journal.pone.0069629 (PMC3724942; doi:10.1371/journal.pone.0069629)
Supplement: Figure S2 — Extracted ion chromatograms of 5 GAs with different linear velocity. (DOC) [file pone.0069629.s012.doc]

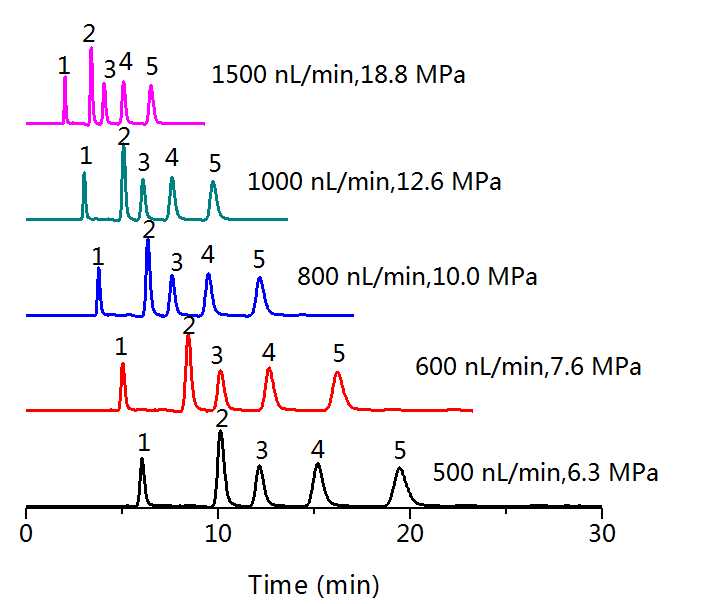


**Figure S2.** Extracted ion chromatograms of 5 GAs with different linear velocity. Experimental conditions: column, poly(META-*co*-DVB-*co*-EDMA) monolithic column (30-cm long, 100 μm *i.d.*, 360 μm *o.d.*); mobile phase, ACN/H_2_O/FA (60/40/0.6, v/v/v). Order of peaks: 1. GA_1_, 2. GA_20_, 3. GA_53_, 4. GA_4_, 5. GA_9_.
