# Supplementary material for: Assessing Gibberellins Oxidase Activity by Anion Exchange/Hydrophobic Polymer Monolithic Capillary Liquid Chromatography-Mass Spectrometry
Source: PLoS One. 2013 Jul 26;8(7):e69629. doi: 10.1371/journal.pone.0069629 (PMC3724942; doi:10.1371/journal.pone.0069629)
Supplement: Figure S3 — Full-scan spectra of GAs under optimized ESI source conditions. (DOC) [file pone.0069629.s013.doc]

**
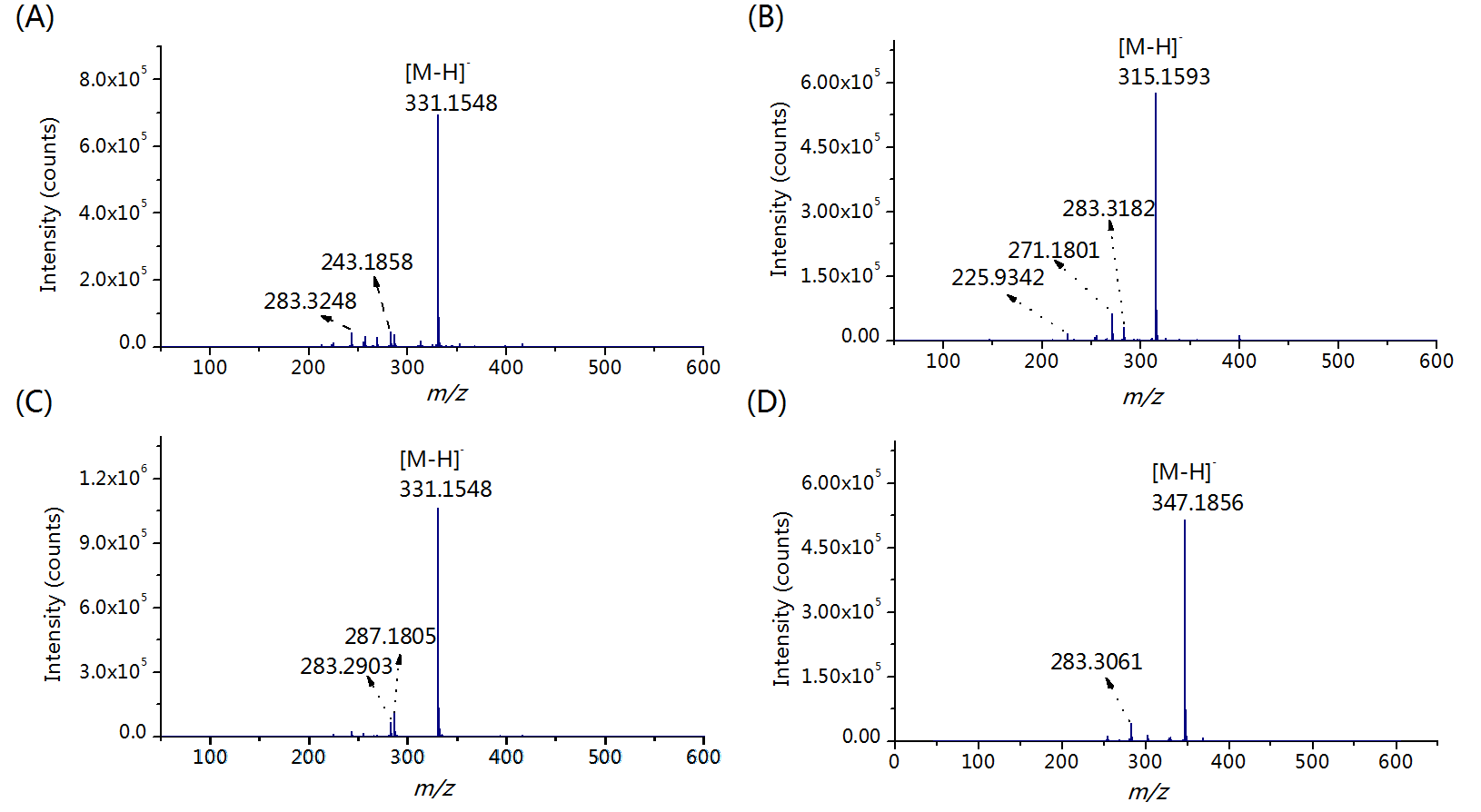
**

**Figure S3.** Full-scan spectra of GAs under optimized ESI source conditions. (A) GA_4_; (B) GA_9_; (C) GA_20_; (D) GA_53_. Experimental conditions: 1 μg/mL GA was infused in mobile phase (ACN/H_2_O/FA, 60/40/0.6, v/v/v) at a flow rate of 3 μL/min.
