# Supplementary figures and images for: Assessing Gibberellins Oxidase Activity by Anion Exchange/Hydrophobic Polymer Monolithic Capillary Liquid Chromatography-Mass Spectrometry
Source: PLoS One. 2013 Jul 26;8(7):e69629. doi: 10.1371/journal.pone.0069629 (PMC3724942; doi:10.1371/journal.pone.0069629)

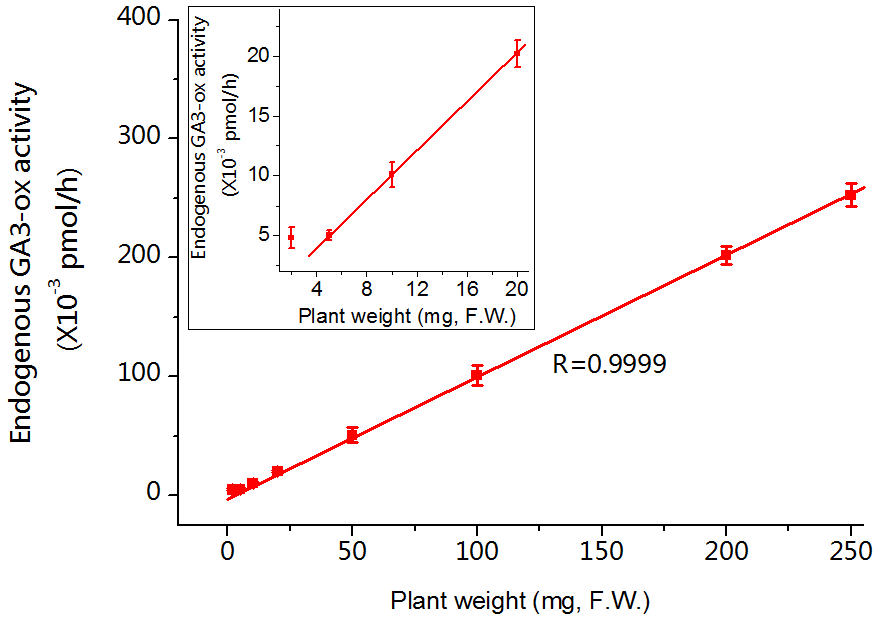


**Figure S4.** The linearity of endogenous GA3-oxidase activity (pmol/h) with different plant weight (mg).

Supplement: Figure S4 — The linearity of endogenous GA3-oxidase activity (pmol/h) with different plant weight (mg). (DOC) [file pone.0069629.s014.doc]
